# Technology for evaluating the ability of probiotic strains to accumulate copper ions *in vitro* and *in vivo* using *Bacillus* bacteria

**DOI:** 10.14202/vetworld.2021.1752-1759

**Published:** 2021-07-06

**Authors:** Aleksey Sizentsov, Elena Salnikova, Yaroslav Sizentsov, Sergey Peshkov, Elena Barysheva, Olga Naumenko, Natalia Romanenko

**Affiliations:** Department of Biochemistry and Microbiology, Orenburg State University, 13, Pobedy Prospect, Orenburg, 460018, Russia

**Keywords:** *Bacillus*, bioaccumulation, copper, *in vitro*, *in vivo*, intoxication, ions, probiotics, technology

## Abstract

**Background and Aim:**

Microorganisms of the genus *Bacillus* comprising probiotics could have an antitoxic effect that is manifested in the active excretion of toxic substances from the body, as well as heavy metals. This study was conducted to assess the effects of copper ions on probiotic strains based on the members of the genus *Bacillus in vitro* and *in vivo*.

**Materials and Methods:**

The following probiotic preparations were selected for this experiment: “Sporobacterin,” “Bactisubtil,” and “Vetom-2.” Sporobacterin liquid, manufactured by Bakoren LLC, is based on *Bacillus subtilis* 534. Bactisubtil, manufactured by Marion Merrell Dow, Inc., is based on *Bacillus cereus* 5832. The first part of the experiment (*in vitro*) was conducted to assess the influence of copper ions on the growth and bioaccumulating ability of probiotic strains. The second part of the experiment (*in vivo*) was conducted to assess the influence of copper ions on the body of laboratory animals and to determine the ability of probiotic strains to remove copper ions from the body of laboratory animals. Statistical analysis was performed using Statistical Package for the Social Sciences, calculating the average value (M), standard deviation (s), and standard deviation error (m). p<0.05 was used to denote statistical significance.

**Results:**

In the previous studies, we found the presence of pronounced sorption characteristics of representatives of both the intestinal microbiome and probiotic strains based on them. In this study, we have studied the prospects of using physiological and adaptive mechanisms of resistance of transient probiotic strains in the system of correction of the elemental status of the animal body due to excessive intake of copper ions into the body. The advantage of their use is due to not only sorption but also the high levels of elimination of complexes accumulated on the surface. Analyzing the data, we can state the following. The excessive content of copper ions inhibits the activity of all microorganisms, and the presence of CuSO_4_ in the nutrient medium reduces the exponential growth phase by 6 h for *B. licheniformis*. The analysis of data on the bioaccumulating property of the probiotic strains under study shows that *B. cereus* (part of Bactisubtil) had the most pronounced sorbing effect with the level of accumulation of 23.96%.

**Conclusion:**

We found that probiotic preparations do not affect biochemical indices of blood and biotissues (the muscle and bone tissue, and the cutaneous covering). As a result of determining the ability of *Bacillus* bacteria comprising the probiotics under this study to accumulate heavy metals by measuring their concentration in the tissues of laboratory animals, the preparations contribute to reducing the toxic effects of copper ions on the body. The cutaneous covering has the greatest accumulation property relative to copper ions. The most effective probiotic under this study in copper ion poisoning was Bactisubtil, and the least effective was Vetom-2.

## Introduction

Probiotics are living microorganisms that are part of the normal microflora of the human intestinal tract. These are nonpathogenic, beneficial bacteria and yeasts that have resistance to opportunistic or pathogenic microorganisms [1-3]. Metals have the ability of bioaccumulation, which is absent in other elements. Furthermore, microorganisms could extract and concentrate metals. This ability is also characterized for microorganisms that are included in probiotic preparations, particularly bacteria of the genus *Bacillus*. In this respect, studying the ability of *Bacillus* bacteria included in probiotics to accumulate heavy metals is important to assess the effectiveness of probiotics in recovery from poisoning caused by heavy metals. In the future, this can serve as a basis for improving probiotic preparations for treating and preventing intestinal infections while simultaneously removing toxic substances, particularly heavy metals, from the body [4-6].

Copper is one of the most important elements needed for living organisms. It is a vital element that is part of many vitamins, hormones, enzymes, and respiratory pigments and involved in metabolic processes and tissue respiration [7]. However, an excessive amount of copper compounds in the body is toxic to humans. Simultaneously, intoxication can be both acquired and hereditary [8]. Note that *Bacillus* bacteria contained in probiotics are self-eliminating antagonists and could have an antitoxic effect that is manifested in the active excretion of toxic substances, particularly heavy metals, from the body [9].

This study was conducted to assess the effects of copper ions on probiotic strains based on the members of the genus *Bacillus in vitro* and *in vivo*.

## Materials and Methods

### Ethical approval

Animals maintenance and procedures during the experiments met the requirements of the instructions and recommendations of Russian regulations (Order of the Ministry of Health of the USSR No. 755 of 12.08.1977) and “The Guide for Care and Use of Laboratory Animals” (National Academy Press, Washington, D.C., 1996).

### Study period and location

The study was conducted in September 2015 at Orenburg State University, Russia.

### Probiotic strains

The following probiotic preparations were selected for our experiment: “Sporobacterin,” “Bactisubtil,” and “Vetom-2.” Sporobacterin liquid, manufactured by Bakoren LLC (Orenburg, Russia), is based on *Bacillus subtilis* 534. Bactisubtil, manufactured by Marion Merrel Dow, Inc. (France), is based on *Bacillus cereus* 5832. Furthermore, Vetom-2 is a bi-preparation manufactured by LLC SPC Issledovatelsky Tcentr (Novosibirsk, Russia) and contains immobilized, dried spore biomass of *B. subtilis* 7048 and *Bacillus licheniformis* 7038. Copper ions (copper sulfate (II)) were used as the toxicant.

### *In vitro* methods

The first part of the experiment (*in vitro*) was conducted to assess the influence of copper ions on the growth and bioaccumulating ability of the probiotic strains under study. The following methods were chosen for the *in vitro* analysis: The serial dilution method, photoelectrocolorimetric method, and atomic-absorption method. Concentrations of the metal salt in test tubes where heavy growth was recorded in the form of flake-shaped sediments (compared with the microorganism growth control) were considered as not affecting the growth of the microorganisms under study. A dilution characterized by a meager increase in the growth of the bacteria under study was marked as the minimum inhibitory concentration (MIC). This technique is described in more detail in the article [10]. The growth dynamics of probiotic cultures were assessed using the optical density of the suspension. The optical density of the bacterial suspensions was measured at a wavelength of 600 nm, in a pan with an optical path length of 10 mm (FEK KFK-3, Russia). The cell concentration in the medium is directly proportional to the value of light diffusion. The optical density of the bacterial suspension was measured at 3-h intervals. The onset of the stationary growth phase was considered three roughly equal values. Thermostatization was held at 37°C. The samples were initially processed according to the standard analysis method using an atomic-adsorption photometer to determine the amount of accumulated metal. A type MGA-915 (MГA-915) device was used as the atomic-absorption spectrophotometer. The effects of metals on the morphology of microbial cells were investigated using atomic-force microscopy. The bacteria were cultivated in a liquid nutrient medium with metal salts for 36 h at 37°C and then were laundered. The concentration of metal salts in which microorganisms were cultured was chosen based on the MIC. The samples were scanned on a CMM-2000 atomic-force microscope (CJSC “PROTON-MIET,” Russia) using the contact mode. The standard software of the microscope was used to analyze the derived images.

### *In vivo* methods

The second part of the experiment (*in vivo*) was conducted to assess the influence of copper ions on the body of laboratory animals and to determine the ability of probiotic strains to remove copper ions from the body of the laboratory animals. Five groups – two control groups and three experimental groups – were formed to conduct the study. The first control group received a basic diet (K0), whereas the second group received a basic diet with copper sulfate (150 mg/kg of body weight) (K2). The three experimental groups received a basic diet with copper sulfate and probiotics: Vetom-2 (O4), Sporobacterin (O5), and Bactisubtil (O6). Metal salts were administered on the 1^st^ day of the experiment, and the probiotics were administered from the 1^st^ day to the 7^th^ day. Biological materials were taken at a frequency of 7 days (the background study, on the 7^th^, 14^th^, and 21^st^ days) by slaughtering the animals using the decollation method. In total, the duration of the experiment was 21 days.

The following methods were used *in vivo*: Measurement of biochemical blood indices, determination of the number of bacteria in the blood and feces of the laboratory animals, and the atomic-absorption method. The derived data were statistically analyzed using Student’s t-test.

## Results

**Figure-1:**
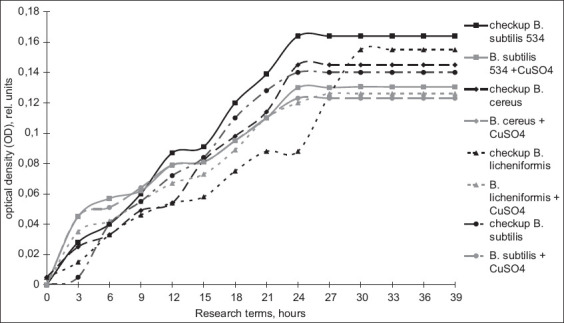
Evaluation of the impact of copper ions on the growth phases of the microorganisms.

The first phase included determining the MICs of copper ions on the growth of the *Bacillus* bacteria contained in probiotic preparations, which allowed us to obtain various concentrations of copper sulfate solutions from the initial 0.02 M. Obtaining several metal solution dilutions were necessary to determine the concentration that has a bactericidal and bacteriostatic effect on the studied microorganisms and concentrations that have no effect on their growth. This will create optimal conditions for cultivating the microorganisms in the presence of metal salt. From the obtained data, it appears that the most sensitive of the strains under study was *B. cereus*, which showed growth suppression at concentrations above 0.0001 M. The remaining microorganisms had growth suppression at concentrations above 0.003 M. At the next stage, we evaluated the effects of copper ions on the growth of the microorganisms under study (Figure-1).

**Figure-2:**
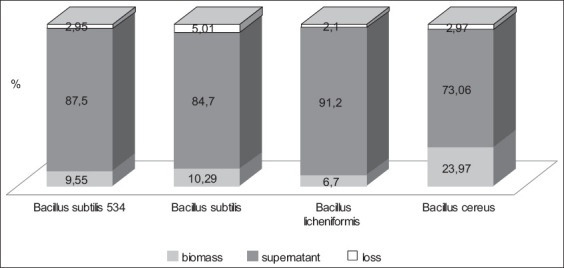
Evaluation of the bioaccumulating property of probiotic strains.

**Figure-3:**
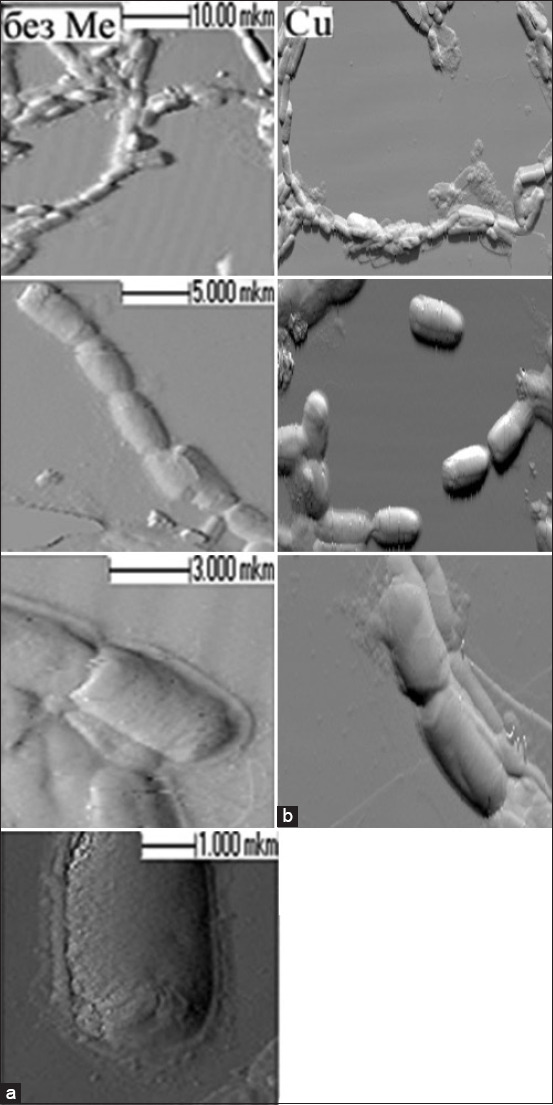
Images of *Bacillus licheniformis 7038* bacteria derived using atomic-force microscopy: (a) No metals; (b) in the presence of copper salts.

The presence of the excessive content of copper ions inhibits the activity of all microorganisms, and copper sulfate reduced the exponential growth phase by 6 h for *B. licheniformis*. The analysis of data on the bioaccumulating property of the probiotic strains under study (Figure-2) showed that *B. cereus* (part of “Bactisubtil”) had the most pronounced sorbing effect at the accumulation level of 23.96%. *B. subtilis*, which is part of the preparations Sporobacterin and Vetom-2, had close accumulation values of 10%, which may indicate the species resistance of this microorganism. *B. licheniformis* (part of Vetom-2) had the lowest sorptive properties of all microorganisms under this study with an accumulation value of 6.7%. *B. licheniformis* 7038 was selected for cell morphology analysis by atomic-strength microscopy. Some bacteria had irregularly shaped particles on the surface (Figure-3). Thus, these may be organic complexes with the metal formed by the release of biomolecules into the nutrient medium by cells. These observations characterize qualitative changes. Quantitative changes are represented in Table-1 and indicate a decrease in the size of bacterial cells.

**Table-1:** Morphological characteristics of *B. licheniformis* 7038.

	Length, μm	Width, μm	Height, nm
К^1^	3.9±0.3	2.2±0.1	513±21
Cu	3.3±0.7	1.8±0.3*	653±104

^1^ К – control without metal,

* p≤0.01

*B. licheniformis*=*Bacillus licheniformis*

**Figure-4:**
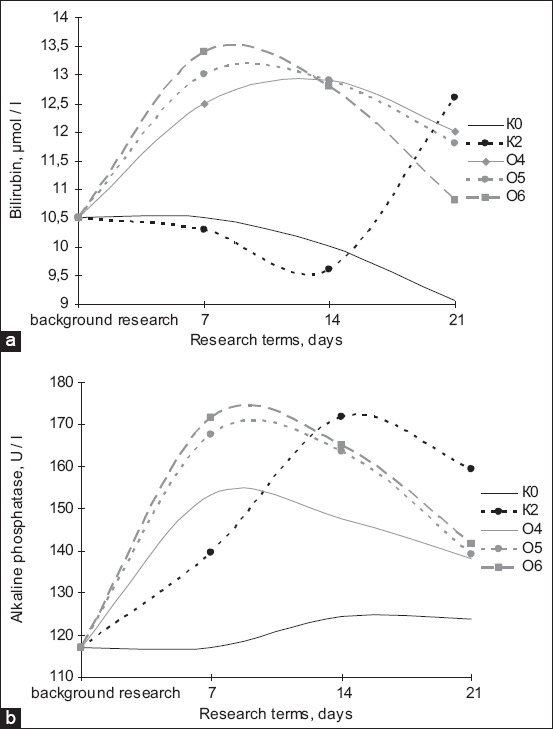
Dynamics of changes in the concentration of total (a) bilirubin and (b) alkaline phosphatase in laboratory animals: K_0_ – background, K_2_ – control of the metal (Cu), O_4_ – “Vetom-2” and caopper, O_5_ – “Sporobacterin” and copper, O_6_ – “Bactisubtil” and copper.

**Figure-5:**
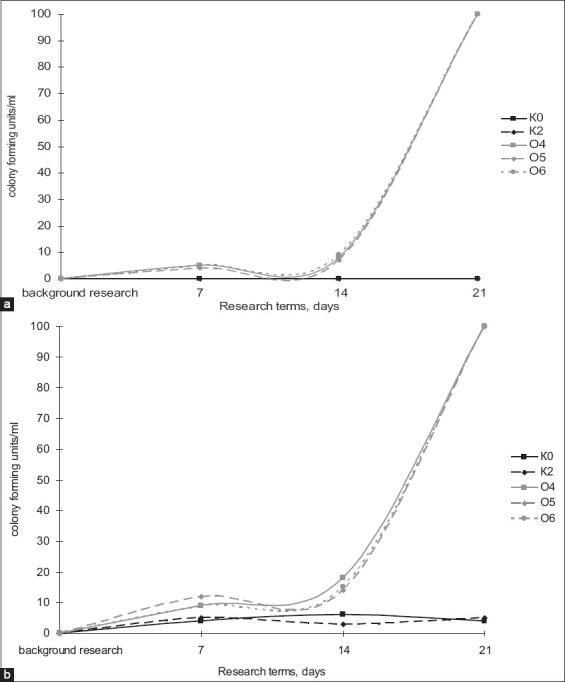
The total number of bacteria of the genus *Bacillus* in (a) blood and (b) feces of laboratory animals: K_0_ – background, K_2_ – control of the metal (Cu), O_4_ – “Vetom-2” and copper, O_5_ – “Sporobacterin” and copper, O_6_ – “Bactisubtil” and copper.

In the second part of the experiment, we studied the following parameters: The *Bacillus* bacterial count in the blood and feces of the laboratory animals (Figure-4), serum biochemical indices (Figures-4 and 5), and the bioaccumulating ability of the *Bacillus* bacteria under study (Tables-2 and 3). Based on the determination of the *Bacillus* bacteria count in the blood of the laboratory animals (Figure-5) relative to background values in K2 on the 14^th^ day, the *Bacillus* bacterial count was 2. Compared with background values, the three experimental groups showed an increase of 15%, 25%, and 20%, respectively, on the 7^th^ day, and an increase was observed on the 14^th^ and 21^st^ days of the study, whereas no increases in K2 and the overall control group were observed.

**Table-2:** The significance of changes in the values of the experimental and control groups compared to K_0_ and K_2_ in the muscle and bone tissues, as well as the cutaneous covering, (μg/g).

Group	Background	After 7 days	After 14 days	After 21 days

The Cu content in the cutaneous covering				
К_0_	0.26±0.05	0.230±0.003	0.22±0.05	0.22±0.03
К_2_	0.37±0.01	0.41±0.08	0.4±0.02	0.58±0.05
О_4_	^••^0.28±0.08*	^••^0.3±0.01**	^••^0.27±0.01*	^•••^0.27±0.01*
О_5_	^••^0.24±0.005*	^••^0.32±0.008**	^••^0.27±0.01*	^•••^0.24±0.005*
О_6_	^••^0.25±0.008*	^••^0.34±0.003***	^•^0.39±0.006***	^•••^0.3±0.005*

**The Cu content in the muscle tissue**				

К_0_	0.24±0.01	0.23±0.03	0.22±0.006	0.22±0.06
К_2_	0.35±0.005	0.4±0.01	0.45±0.01	0.48±0.008
О_4_	^•••^0.25±0.003*	^•^0.46±0.008**	^•^0.37±0.003***	^•••^0.34±0.006*
О_5_	^•••^0.23±0.008*	^•^0.35±0.008*	^••^0.32±0.008**	^•••^0.32±0.008*
О_6_	^••^0.25±0.008*	^•^0.43±0.005*	^•^0.39±0.01**	^••^0.37±0.003*

**The Cu content in the bone tissue**				

К_0_	0.21±0.01	0.23±0.003	0.22±0.01	0.22±0.003
К_2_	0.23±0.01	0.58±0.01	0.6±0.02	0.64±0.01
О_4_	^•^0.2±0.01*	^•••^0.38±0.008***	^••^0.34±0.03*	^•••^0.31±0.008**
О_5_	^•^0.22±0.01*	^••^0.47±0.01***	^••^0.45±0.006***	^•••^0.44±0.01***
О_6_	^•^0.2±0.008*	^•^0.44±0.03**	^•••^0.3±0.01**	^•••^0.26±0.003**

* р < 0.5;

** р < 0.05;

*** р < 0.005 сompared to К_0_^•^р < 0.5; ^••^р < 0.05; ^•••^р < 0.005 сompared to К_2_

**Table-3:** Dynamics of copper removal with probiotic preparations *in vivo* (on the 21^st^ day of the experiment) and *in vitro in %.*

Probiotic preparations	Biological materials	*in vivo*		*in vitro*

		Removal from tissues	Mean	
“Sporobacterin”	Muscle tissue	29.16	44.72	10.0
	Bone tissue	51.56		
	Cutaneous covering	53.44		
“Vetom-2”	Muscle tissue	33.33	37.7	16.7
	Bone tissue	31.25		
	Cutaneous covering	48.52		
“Bactisubtil”	Muscle tissue	53.75	57.13	23.96
	Bone tissue	59.37		
	Cutaneous covering	58.27		

**Figure-6:**
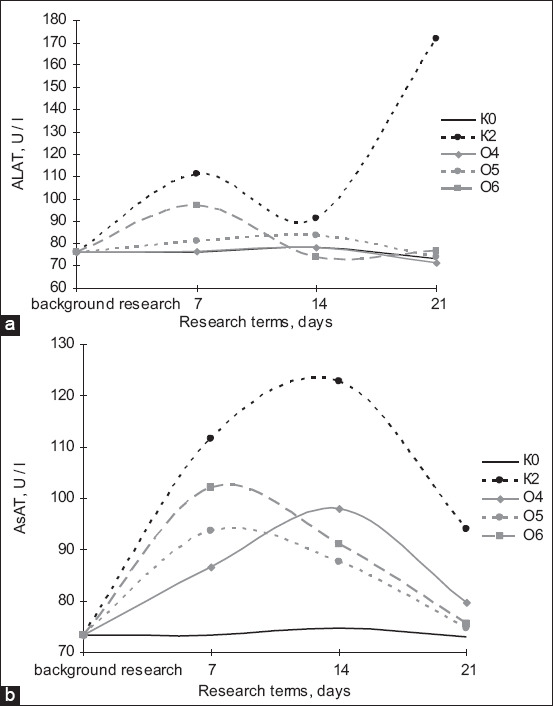
Dynamics of changes in the concentration of (a) ALT and (b) AST in laboratory animals: K_0_ – background, K_2_ – control of the metal (Cu), O_4_ – “Vetom-2” and copper, O_5_ – “Sporobacterin” and copper, O_6_ – “Bactisubtil” and copper.

Figure-4 shows a change in the *Bacillus* bacterial count in the feces of the laboratory animals. Furthermore, in relation to background values on the 7^th^ day of the study, the experimental groups had an increase of 66.6%, 100%, and 66.6% in *Bacillus* bacterial count. The effects of probiotic preparations and copper on biochemical blood indices (i.e. total bilirubin, alkaline phosphatase, alanine aminotransferase [ALT], and aspartate aminotransferase [AST]) throughout the study were examined. To visualize the data in dynamics, we built graphs for these biochemical indices. The analysis of the total bilirubin dynamics (Figure-6a) allowed us to generalize the overall dynamics of the experimental groups on the 7^th^ day of the study as the values of all three experimental groups were comparable, whereas the concentration of this index in K2 was below the level of the overall control group. On the 14^th^ day of the study, the total bilirubin values of the experimental groups varied widely with O4 having the highest value and O6 having the lowest value when K2 had the lowest values at this stage of the study. On the 21^st^ day of the study, O5 and O6 had a decrease in total bilirubin concentrations in the serum, but its concentrations in O4 and O5 were significantly higher than that of the overall control group; however, on the final stage of the study, the highest total bilirubin concentration was recorded in K2.

One of the criteria blood biochemical indices for treatment effectiveness is serum alkaline phosphatase concentration (Figure-6b). The data presented in the graph show the effectiveness of probiotic preparations based on *Bacillus* bacteria in treating copper intoxication. On the 21^st^ day, the serum alkaline phosphatase concentration in all experimental groups decreased, and Sporobacterin (used in O5) appeared to be the most effective preparation because its values were the closest to K0 throughout the study. The dynamics of ALT concentration (Figure-6a) indicated that on the 7^th^ day of the study, all groups that received copper sulfate as the toxicant had significantly higher concentrations than the overall control with O6 having the highest ALT content and O4 having the lowest values. On the 14^th^ day of the study, the highest serum ALT concentration was observed in O5, whereas O6 had the lowest values. On the 21^st^ day of the study, O5 showed a decrease to the lowest values among the experimental groups; the ALT level in O4 slightly decreased compared with that reported in the previous study, but it should be noted that the metal intoxication control group recorded a sharp increase in ALT concentrations, far exceeding the indices not only of the overall control group but also of all control samples.

The analysis of serum AST concentrations of the experimental animals on the 7^th^ day of the study found that AST had higher values than that of the overall control group relative to group K0. On the 14^th^ day of the study, the difference in AST compared with K0 was 16.6%, 31.3%, 17.4%, and 22.0% for K2, O4, O5, and O6, respectively. On the 21^st^ day of the study, even higher AST concentrations compared with K0 were observed: The difference was 8.21%, 9.04%, 2.19%, and 3.56% for K2, O4, O5, and O6, respectively. In relation to the copper intoxication control group, all groups recorded values above those of the control group with differences of 0.75%, 5.5%, and 4.3% for O4, O5, and O6, respectively.

Generalizing the data provides an opportunity to state the effectiveness of the use of probiotic preparations based on *Bacillus* bacteria in treating copper intoxication, as the main biochemical parameters allowing the evaluation of the metabolism processes related to detoxification, which include total bilirubin, ALT, and alkaline phosphatase, were lower than those of the copper intoxication control group on the 21^st^ day of the study, and the overall control group had minor deviations. The ability of *Bacillus* bacteria contained in probiotics to remove copper from the body was evaluated using the metal concentrations in the tissues of laboratory animals. The following biological materials were investigated for this purpose: Bone and muscle tissues and the cutaneous covering of the laboratory animals. Using atomic-absorption spectrophotometry, copper concentrations in the tissues under study (Table-2) were measured.

The experiment found that the copper concentrations in the bone tissue of the laboratory animals on the 7^th^ day of the study in the experimental groups (i.e. O4, O5, and O6) exceeded those in K0 by 65%, 99%, and 91%, respectively; however, the copper level in K2 was lower than that in O4 by 34%, in O5 by 18%, and in O6 by 24%. Then, a steady decrease in the copper levels was observed on the 14^th^ and 21^st^ days of the experiment with the 14^th^-day values being below the 7^th^-day ones by 10%, 4%, and 31% in O4, O5, and O6, respectively. These indices remained higher in K2; that is, K2 exceeded O4 (14^th^ day) by 43%, O5 by 25%, and O6 by 50% on the 14^th^ day of the study.

On the 21^st^ day of the experiment, the copper contents were lower than those on the 14^th^ day by 8%, 2%, and 13%, respectively. Note that the copper levels in K2 were even higher than those in O4 by 51%, O5 by 31%, and O6 by 59% on the 21^st^ day of the experiment. The copper content in the muscle tissue of the laboratory animals on the 7^th^ day in the experimental groups (i.e. O4, O5, and O6) exceeded those in K0 by 99%, 52%, and 86%, respectively; however, the levels of the indices in the experimental groups were lower than those in K2 by 15% versus O4, by 12% versus O5, and by 7% versus O6. On the 14^th^ day, the copper levels in K2 decreased compared with those in the experimental groups on the 7^th^ day by 19% versus O4, by 8% versus O5, and by 9% versus O6, and therefore, the difference between the experimental groups and K2 increased by 2%, 16%, and 6%, respectively. Regarding the removal of copper from bone tissue on the 21^st^ day of the experiment, a decrease in copper levels removed from the muscle tissue was observed, and the copper content decreased in the experimental groups on the 21^st^ day of the experiment compared with those on the 14^th^ day by 8%, 1%, and 5%, respectively. On day 21, the copper levels in K2 exceeded those in O4 by 29%, O5 by 33%, and O6 by 22%.

The copper content in the cutaneous covering of the laboratory animals on the 7^th^ day of the experiment exceeded those in K0 by 30% in O4, by 39% in O5, and by 47% in O6, whereas these values remained lower than those in K2 by 27%, 22%, and 17%, respectively. The values in the experimental groups on the 14^th^ day of the experiment were lower than those on the 7^th^ day by 10%, 15%, and 14%, respectively, and the difference between K2 and the experimental groups increased by 5%, 10%, and 3%, respectively. On the 21^st^ day of the experiment, a reduction in the copper removal was observed; the 21^st^-day values in the experimental groups differed from 14^th^-day ones by 1% for O4, by 10% for O5, and by 20% for O6. On the 21^st^ day of the experiment, the values in K2 were higher than those in the experimental groups by 53%, 59%, and 48%, respectively.

The results are not in complete accordance with the results derived from the *in vitro* experiment where the least active biosorbent was Sporobacterin, whereas *in vivo*, it was Vetom-2 (Table-3). However, both experiments proved that *B. cereus* 5832, which is part of Bactisubtil, was a more active biosorbent.

## Discussion

In the previous studies, we found pronounced sorption characteristics of representatives of both the intestinal microbiome and probiotic strains based on them. In this study, we have studied the prospects of using physiological and adaptive mechanisms of resistance of transient probiotic strains in the system of correction of the elemental status of the animal body due to excessive copper intake. The advantage of their use is due to not only sorption but also the high level of elimination of complexes accumulated on the surface. The use of the biological potential of representatives of the microbiome and transient symbiotic flora is essential to the scientific community in various fields of human activity. Along with the classical idea of the physiological significance of probiotic strains in the processes of human and animal life, some studies have aimed at studying alternative ways to use the antagonistic and physiological-adaptive characteristics of bacterial strains [11-13]. Considering the updated data, updated ideas are formed about the participation of representatives of the microbiome and probiotic strains, particularly in physiologically significant processes [14-16].

The generalized analysis of experimental data allows us to state, with a high level of confidence, the presence of mechanisms of sorption of active copper cations by the strains under study, which is consistent with the previously published data on the presence of sorbing characteristics of *Bacillus* bacteria with respect to heavy metals [17]. The high level of biotoxicity of copper cations relative to the microorganisms under this study and laboratory animals in relatively high concentrations may be due to the lack of detoxification mechanisms of protection against essential elements [18]. This explains the relatively low level of cation sorption in *in vitro* experiments and the presence of high cation load reduction characteristics in the tissues of the experimental animals. The advantage of using probiotic strains based on *Bacillus* bacteria is their ability to sorb excessive concentrations of active metal cations not only in the intestinal lumen but also in the body tissues, reducing the degree of hepatotoxic effect, which is confirmed by the results of the biochemical study of the blood of the experimental animals [19-21].

## Conclusion

This study was conducted to examine the effectiveness of probiotic preparations based on spore-forming *Bacillus* bacteria in the experimental intoxication of laboratory animals with copper. In this study, we found that probiotic preparations based on spore-forming *Bacillus* bacteria do not affect biochemical indices of blood and biotissues (i.e. the muscle and bone tissue and the cutaneous covering). In addition, the preparations contribute to reducing the toxic effects of copper ions on the body. Thus, the cutaneous covering has the greatest accumulation property relative to copper ions. The most effective probiotic under this study in copper ion poisoning was Bactisubtil, whereas the least effective one was Vetom-2.

## Authors’ Contributions

AS, ES, and YS: Designed the experiment. SP and EB: Performed the experimentation. ON and NR: Wrote and edited the manuscript. All authors have read and approved the final manuscript.
